# Characteristics of Patent Foramen Ovale: Analysis from a Single Center

**DOI:** 10.1155/2022/5430598

**Published:** 2022-04-06

**Authors:** Bin Zhang, Dong Li, Anjian Song, Qiang Ren, Shangan Cai, Peng Wang, Wenfeng Tan, Gaoxing Zhang, Jun Guo

**Affiliations:** ^1^Department of Cardiology, The First Affiliated Hospital of Jinan University, Guangzhou 510630, China; ^2^Department of Cardiovascular Disease and Clinical Experimental Center, Jiangmen Central Hospital, Jiangmen 529030, China; ^3^Department of Intensive Care Unit and Clinical Experimental Center, Jiangmen Central Hospital, Jiangmen 529030, China; ^4^Department of Urology, Jiangmen Central Hospital, Jiangmen 529030, China; ^5^Department of Information, Jiangmen Central Hospital, Jiangmen 529030, China

## Abstract

**Objective:**

To collect and analyze data of patent foramen ovale (PFO).

**Methods:**

This study included a total of 260 patients diagnosed with PFO. We analyzed basic clinical data such as sex, age, transesophageal echocardiography as well as other symptoms.

**Results:**

Our data showed that females accounted for the highest proportion of PFO (166 females, 64%), with the highest number of patients (65 patients) having between 45 and 55 years. Transesophageal echocardiography examination demonstrated frequent occurrence of tunnel-like anatomical structures. In addition, PFO was associated with symptoms such as migraine, stroke or TIA, syncope, chest tightness, and palpitations, with dizziness being the most common symptom in the patients with PFO.

**Conclusion:**

Our data demonstrated that females accounted for the highest proportion of PFO patients, with those aged between 45 and 55 years being most affected. The most frequently encountered clinical symptom was dizziness. Taken together, these findings may help doctors to better understand and screen for PFO patients.

## 1. Introduction

Foramen ovale is the passage that connects the left and right atria in the fetus. Before birth, the fetus does not breathe through the lungs. Oxygen-carrying maternal blood enters the left atrium from the right atrium through the foramen ovale, which is then supplied to all parts of the body. After birth, there is oxygen filling of the alveoli which expands the pulmonary arterioles, leading to suppression of pulmonary circulation resistance and right atrial pressure. At the same time, increase in return blood volume after the pulmonary circulation leads to an increase in the left atrial pressure, which together promote the closure of the foramen ovale [1–3]. Under normal circumstances, the foramen ovale is closed within the first year of life. Lack of closure of the foramen ovale at the age of 3 years leads to patent foramen ovale (PFO). About 20–25% of adults have incomplete foramen ovale closure [4–8].

Previous data showed that PFO had little effect on cardiac hemodynamics and could be left untreated. However, recent studies have demonstrated that the presence of PFO is closely associated with occurrence of paradoxical embolism, stroke, migraine, and other diseases. Besides, presence of atrial septal herniation with PFO is a risk factor for cerebral infarction [2–4, 8–13]. Therefore, many studies have analyzed the role of PFO on stroke and migraine [10, 14–19]. However, PFO can also lead to other symptoms which include dizziness, syncope, chest tightness, and palpitations [20–28]. Therefore, it is important to characterize and understand the clinical characteristics of PFO. In this study, we collected and analyzed clinical data of PFO patients in our center. Findings of this study might help doctors to better understand and screen for PFO patients.

## 2. Materials and Methods

A total of 260 patients diagnosed with PFO patients were included in this study. The patients underwent transesophageal echocardiography, the mainstay diagnostic tool for PFO [29]. We collected and analyzed the basic clinic information including sex, age, transesophageal echocardiography, and associated symptoms to characterize the PFO. This study was approved by the Administrative Committee of Experimental Animal Care and Use of our hospital.

### 2.1. Gender Composition Analysis

We analyzed the number and proportion of males and females in the 260 enrolled PFO patients.

### 2.2. Age Distribution

The patients were divided into different groups according to their age, at an interval of 10 years (＜15 years old, 15–25 years old, 25–35 years old, 35–45 years old, 45–55 years old, 55–65 years old, 65–75 years old). Age distribution was used to demonstrate the predisposing or common population in PFO.

### 2.3. Anatomical Classification Based on Transesophageal Echocardiography

Patients undergoing transesophageal echocardiography with sedation are required to abstain from food and beverages (other than clear liquids) for a minimum of 6 hours before the planned procedure and restrain from all intake for 3 hours before the procedure [29, 30]. Patients with delayed gastric emptying and other aspiration risks may need a longer period of fasting or preprocedural administration of agents, such as metoclopramide, to minimize the risk for residual gastric contents and aspiration [29]. The patients first used dyclonine for local throat anesthesia and then inserted it with an ultrasound probe for examination. Based on the transesophageal echocardiography results, anatomical classification was divided into long-tunnel, crevice-like, and other types. Furthermore, they were divided into those with or without aneurysmal septum. The number and proportion of the different types were analyzed.

### 2.4. Symptom Analysis

The 260 PFO patients were interviewed and recorded, and 5 kinds of symptoms: dizziness, migraine, syncope, stroke or TIA, chest tightness, and palpitations were identified. The number and proportion of these five types of symptoms were analyzed.

## 3. Results

### 3.1. Gender Composition

**Figure 1:**
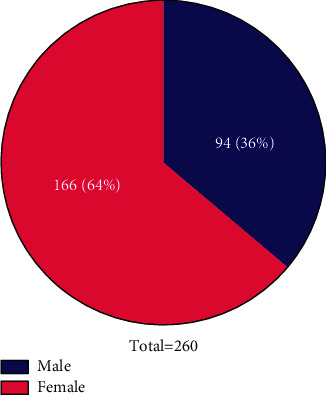
Gender composition. In the pie chart, blue represents men and red represents women. White font represents the number and percentage of each part.

Out of the 260 PFO patients, 166 were females, which accounted for 64% while 94 (36%) were males (Figure 1).

### 3.2. Age Distribution

**Figure 2:**
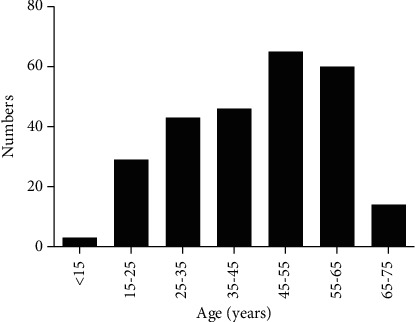
Age distribution. The histogram represents the number and distribution of patients in different age groups.

The analysis showed that there were 5 PFO patients for age＜15 years old, 29 for 15–25 years old, 43 for 25–35 years old, 46 for 35–45 years old, 65 for 45–55 years old, 60 for 55–65 years old, and 14 for 65–75 years old (Figure 2).

### 3.3. Anatomical Classification Based on Transesophageal Echocardiography

**Figure 3:**
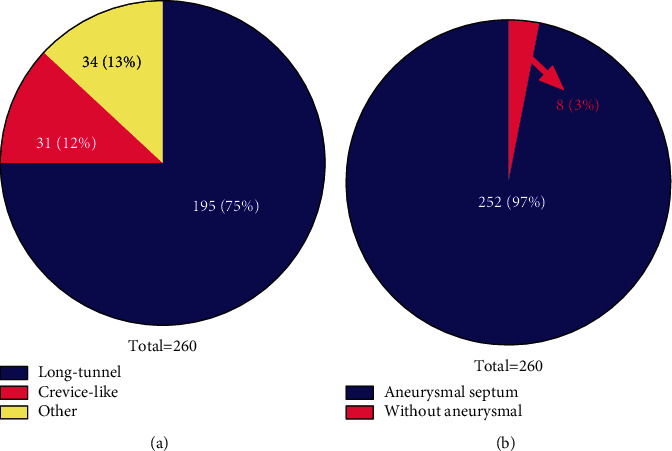
Anatomical classification based on transesophageal echocardiography. (a) Blue represents long-tunnel type, red represents crevice-like type, and yellow represents other type. (b) Red represents accompanied with aneurysmal septum and blue represents without aneurysmal septum.

Out of the total 260 PFO patients, 195 (75%) were long-tunnel type, while 31 were crevice-like type, and 34 included other types, accounting for 36%. 8 PFO patients were accompanied with aneurysmal septum, while 252 were normal without aneurysmal septum (Figure 3).

### 3.4. Symptoms Recorded and Analysis

#### 3.4.1. Dizziness

**Figure 4:**
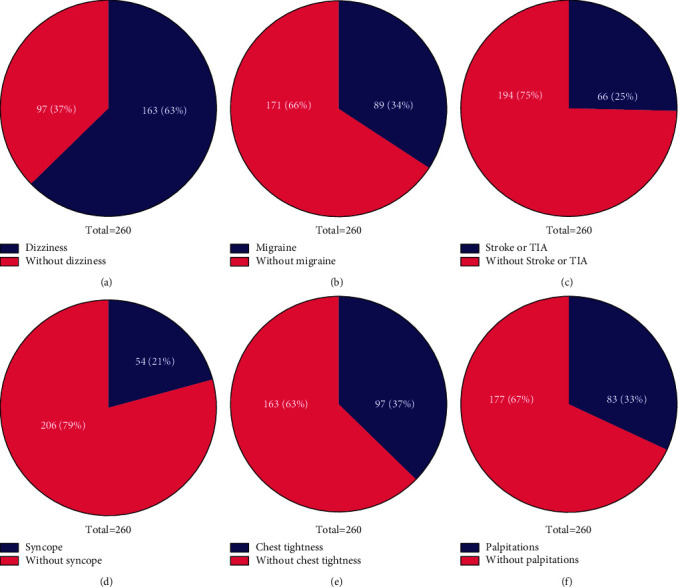
Symptoms recorded and analysis. (a) Blue represents patients accompanied with dizziness while red represents patients without dizziness. (b) Blue represents patients accompanied with migraine while red represents patients without migraine. (c) Blue represents patients accompanied with stroke or TIA while red represents patients without stroke or TIA. (d) Blue represents patients accompanied with syncope while red represents patients without syncope. (e) Blue represents patients accompanied with chest tightness while red represents patients without chest tightness. (f) Blue represents patients accompanied with palpitations while red represents patients without palpitations.

Out of the 260 PFO patients, 163 (63%) were accompanied with dizziness, while 97 (37%) did not experience dizziness (Figure 4(a)).

#### 3.4.2. Migraine

Out of the 260 PFO patients, 89 (34%) had migraine, while 171 (66%) were without migraine (Figure 4(b)).

#### 3.4.3. Stroke or TIA

In the 260 PFO patients, 66 (25%) were accompanied with stroke or TIA, while 194 (75%) did not experience dizziness (Figure 4(c)).

#### 3.4.4. Syncope

Out of the 260 PFO patients, 54 (21%) were accompanied with syncope, while 206 (79%) were without syncope (Figure 4(d)).

#### 3.4.5. Chest Tightness

Out of the 260 PFO patients, 97 (37%) were accompanied with chest tightness, while 163 (63%) did not have chest tightness (Figure 4(e)).

#### 3.4.6. Palpitations

Out of the 260 PFO patients, 83 (33%) were accompanied with palpitations, while the remaining 67% did not have palpitations (Figure 4(f)).

### 3.5. Composition of the Symptoms

**Figure 5:**
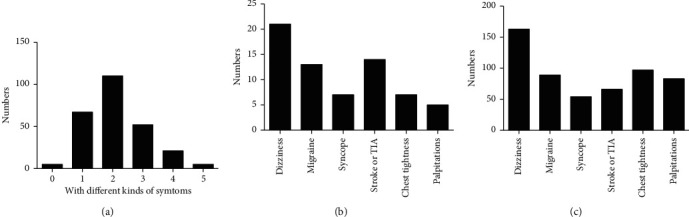
Composition of different kinds of symptoms. (a) The number of patients with different kinds of symptoms. (b) The composition of the 67 patients with only one symptom. (c) The number of patients with different symptoms.

As shown in Figure 5(a), out of the 260 PFO patients, 5 did not have any symptom. 67 patients were accompanied with only one kind of symptom, 110 had 2 kinds of symptoms, 52 were accompanied with 3 kinds of symptoms, and 21 had 4 kinds of symptoms while the remaining 5 were accompanied with 5 kinds of symptoms. Out of the 67 patients with only one symptom, the most common symptoms were dizziness (21 patients), migraine (13 patients), syncope (7 patients), stroke or TIA (14 patients), chest tightness (7 patients), and palpitations (5 patients).  Figure 5(c) shows in all patients, 163 patients had the dizziness, 89 had migraine, 54 had syncope, 66 had stroke or TIA, 97 had chest tightness, and 83 had palpitations.

## 4. Discussion

This study showed that there were more females with PFO in the clinic. Besides, patients aged between 45 and 55 years old had the highest burden of PFO. Analysis by transesophageal echocardiography demonstrated that the most common type of anatomy was the tunnel type, with few having aneurysmal septum. Out of all the symptoms, dizziness was the most common symptom in the patients with PFO.

Previous data indicated that PFO had little effect on cardiac hemodynamics and thus could be left untreated. However, recent studies have shown that PFO is of clinical significance as it is a source of thrombus formation or could be a conduit for paradoxical embolism [31]. Previous reports showed an association between ischemic stroke and PFO because of paradoxical venous thromboembolism [32–35]. In addition, recent analyses have focused on stroke, TIA, or migraine [9, 10, 14–19, 31, 32, 36–41]. The data showed that besides cryptogenic stroke, paradoxical embolism, and migraine, PFO can result in dizziness, syncope, chest tightness, and palpitations. This study showed that the most common symptom was dizziness, stroke, or TIA, as well as migraine. Therefore, there is need to not only pay attention to serious complications such as stroke or TIA but also related symptoms such as dizziness, syncope, chest tightness, and palpitations. These symptoms can seriously affect the quality of life of the PFO patients. On the other hand, it reminds doctors that when they observe the above-mentioned symptoms in the clinic, without other positive from related tests or systems, they should screen for PFO, especially in females between the ages of 15 and 65 years. Since early diagnosis and treatment can improve the patients' symptoms and quality of life and prevent the occurrence of stroke, in sync, this study analyzed the gender, age, and symptoms of the PFO patients to guide the screening and treatment of the PFO patients.

Although this study highlights important findings, it analyzed data from a single center, and thus there is a need for a larger sample size. Besides, we did not evaluate related effects of interventional closure therapy, especially improvement of symptoms other than stroke or TIA.

## 5. Conclusion

Our data showed that females accounted for the highest proportion of PFO patients, and most patients were in the 45–55 age group. PFO was associated with symptoms such as dizziness, migraine, stroke or TIA, syncope, chest tightness, and palpitations. The most frequently encountered clinical symptom was dizziness. Together, characterizing the PFO patients in terms of gender, age, and associated symptoms may help doctors to better understand and screen for PFO.

## Data Availability

All the data used to support the findings of this study are available from the corresponding author upon request.
